# Protocol for a randomised controlled trial of an outreach support program for family carers of older people discharged from hospital

**DOI:** 10.1186/s12877-015-0065-5

**Published:** 2015-06-25

**Authors:** Christine Toye, Rachael Moorin, Susan Slatyer, Samar M. Aoun, Richard Parsons, Desley Hegney, Sean Maher, Keith D. Hill

**Affiliations:** School of Nursing, Midwifery and Paramedicine, Faculty of Health Sciences, Curtin University, GPO Box U1987, Perth, WA 6845 Australia; Centre for Nursing Research, Sir Charles Gairdner Hospital, Perth, WA 6009 Australia; School of Public Health, Curtin University, Perth, WA 6845 Australia; School of Population Health, The University of Western Australia, Perth, WA 6009 Australia; Department of Research, Silver Chain Group, Osborne Park, Perth, WA 6017 Australia; School of Pharmacy, Curtin University, Perth, WA 6845 Australia; School of Nursing and Midwifery, The University of Southern Queensland, Toowoomba, QLD 4350 Australia; School of Nursing, The University of Adelaide, Adelaide, South Australia 5005 Australia; Department of Rehabilitation and Aged Care, Sir Charles Gairdner Hospital, Perth, WA 6009 Australia; School of Physiotherapy and Exercise Science, Curtin University, Perth, WA 6845 Australia

## Abstract

**Background:**

Presentations to hospital of older people receiving family care at home incur substantial costs for patients, families, and the health care system, yet there can be positive carer outcomes when systematically assessing/addressing their support needs, and reductions in older people’s returns to hospital attributed to appropriate discharge planning. This study will trial the Further Enabling Care at Home program, a 2-week telephone outreach initiative for family carers of older people returning home from hospital. Hypotheses are that the program will (a) better prepare families to sustain their caregiving role and (b) reduce patients' re-presentations/readmissions to hospital, and/or their length of stay; also that reduced health system costs attributable to the program will outweigh costs of its implementation.

**Methods/Design:**

In this randomised controlled trial, family carers of older patients aged 70+ discharged from a Medical Assessment Unit in a Western Australian tertiary hospital, plus the patients themselves, will be recruited at discharge (*N* = 180 dyads). Carers will be randomly assigned (block allocation, assessors blinded) to receive usual care (control) or the new program (intervention). The primary outcome is the carer’s self-reported preparedness for caregiving (Preparedness for Caregiving Scale administered within 4 days of discharge, 2–3 weeks post-discharge, 6 weeks post-discharge). To detect a clinically meaningful change of two points with 80 % power, 126 carers need to complete the study. Patients’ returns to hospital and subsequent length of stay will be ascertained for a minimum of 3 months after the index admission. Regression analyses will be used to determine differences in carer and patient outcomes over time associated with the group (intervention or control). Data will be analysed using an Intention to Treat approach. A qualitative exploration will examine patients’ and their family carers’ experiences of the new program (interviews) and explore the hospital staff’s perceptions (focus groups). Process evaluation will identify barriers to, and facilitators of, program implementation. A comprehensive economic evaluation will determine cost consequences.

**Discussion:**

This study investigates a novel approach to identifying and addressing family carers’ needs following discharge from hospital of the older person receiving care. If successful, the program has potential to be incorporated into routine post-discharge support.

**Trial registration:**

Australian and New Zealand Clinical Trial Registry: ACTRN12614001174673.

## Background

This study is being conducted in metropolitan Western Australia (WA), where increasing numbers of older people are experiencing multiple chronic health conditions, disability, and high level health care utilization [1]. This situation parallels that in other western countries in which life expectancy is increasing [2] and, together with predictions of health workforce shortages [3], highlights the need to increase support for family-based home care within the community [4]. There were close to 350,000 informal primary carers of older people in Australia in 2009, saving an estimated $40 billion in service provision costs each year [5] but poor health has long been a recognised family caregiver outcome, with support needed to help carers to sustain their caregiving role [6].

For older people receiving care and in poor health, readmissions to hospital are potentially distressing and costly, yet sometimes avoidable with high quality discharge planning [7]. A key component of effective discharge planning is to closely involve the family carers [8]. However, in a recent study investigating returns to hospital after discharge from a short stay (<72 h) Medical Assessment Unit (MAU), communication issues at the time of discharge presented a significant challenge [9]. There was limited opportunity for the staff to liaise with family carers in the time available prior to discharge. In particular, there was insufficient time allocated to assess carers’ needs and communicate plans to them for addressing likely future health crises for patients [9].

In parallel work in community palliative care in the United Kingdom, a need for a systematic and effective carer needs assessment process was identified. The Carer Support Needs Assessment Tool (CSNAT) was developed following extensive reviews of the literature [10–12], and from interviews with bereaved carers [13]. The tool was subsequently validated using a sample of 225 current carers [14]. The CSNAT is a brief tool, yet covers all identified support need areas. Each item represents a core carer support domain in home care. The items fall into two sets: those that enable the carer to care for the patient at home and those that enable direct support for the carer [13]. The CSNAT has recently been used in a Western Australian study in palliative home care; it was reported to be practical and useful; to prompt carers to consider their own needs rather than just those of the patient; to validate, reassure, and empower carers; and to help carers access the support that they need [15].

The aim of this study is to determine how implementing an evidence-based, systematic, and targeted support program for family carers of older patients - immediately after the patient’s discharge from a metropolitan Western Australian hospital’s MAU - impacts upon these carers; the patients for whom they provide care; and costs to WA Health, the local health care system. The program being evaluated is termed the Further Enabling Care at Home (FECH) program, and it uses the CSNAT to identify and prioritise family carers’ support needs that are subsequently addressed. The study has three hypotheses:

- H1: That the enhanced support of family carers facilitated by the FECH program will prepare families to sustain their caregiving role better than usual care.
- H2: That, because family carer roles are better supported, implementation of this program will reduce (a) patients’ re-presentations and readmissions to hospital within 3 months of discharge, and (b) length of hospital stay when readmissions do occur.
- H3: That reduced costs to WA Health will outweigh costs of implementing the program.

Study objectives are to:

1. Compare family carer preparedness to care between those included in the FECH program and those receiving usual care.
2. Compare patient outcomes from the FECH program versus those from usual care in terms of re-presentations to Emergency Departments (EDs), readmissions to hospital within the months (at least 3) following the index separation date – also Length of Stay (LoS) if readmission does eventuate.
3. Document costs of a suitably qualified nurse (FECH nurse) implementing the program to inform the economic evaluation and determine a sustainable full time FECH nurse ‘caseload’.
4. Compare costs of re-presentations to EDs, hospital readmissions, and LoS between the intervention group (FECH program) and the control group (usual care).
5. Estimate overall cost effectiveness for the local health system from FECH program implementation during the study period.
6. Explore and describe family carers’ perceptions of how the FECH program impacts upon caregiving sustainability plus any other outcomes for them; also how it might be refined to (a) minimise any negative outcomes, (b) enhance those that are positive, and (c) (further) enhance caregiving sustainability.
7. Explore and describe patients’ perceptions of how the FECH program impacts upon them and how it might be refined to ensure the best possible outcomes (for them).
8. Use process evaluation and obtain perspectives of MAU health care professionals to determine how the FECH program’s implementation might be improved.
9. Refine the program from study recommendations and develop a plan to embed it into practice in the study setting and other similar health services.
10. Develop a plan for testing the FECH program in other health settings – should it be shown to have cost benefits and no negative patient/carer outcomes when implemented in the MAU.

## Method

### Trial design

This is a mixed methods study addressing objectives and testing hypotheses in a single blind *Randomised Controlled Trial* (RCT) (concealed allocation), adhering to CONSORT guidelines for transparent reporting [16], during which *Qualitative Evaluation* and FECH program *Process Evaluation* will also be undertaken. The study will also be supported by an expert reference group. The RCT will compare outcomes between the control condition (usual care) and the experimental condition (usual care plus family carer inclusion on the new FECH program). Approval has been received from the Sir Charles Gairdner Group Human Research Ethics Committee (HREC) (2014–133), the Department of Health WA HREC (2014/78), and from the Curtin University HREC (HR14/2015).

### Study setting and participants

The MAU is a 36-bed unit providing intensive assessment and treatment for patients with acute medical conditions, most of whom are over 70 years of age. MAUs are widely implemented in the UK, Australia and New Zealand in response to the need to increase patient through-put [17] and sustained health system pressures suggest that their use will continue to expand. Care is provided by physician-led multidisciplinary teams for up to 72 h, after which patients are discharged or transferred to inpatient units for ongoing management. A key component of the MAU model is that ongoing management for discharged patients is provided through prompt general practitioner follow-up [9].

Included participants will comprise dyads of older people (aged 70+) discharged home from the MAU during the recruitment period and their adult (aged 18+), English speaking, family carers (one per patient). The definition of a family carer is that used in a recent Australian study investigating the care of frail older people: “a family member or friend who provides unpaid personal care, support and assistance” [18]. Dyads already recruited into the study will be excluded from further recruitment upon any subsequent readmissions to the MAU.

### Experimental condition (inclusion in the FECH program)

Carer participants in the intervention group will receive standard care plus the FECH program implemented by the FECH nurse who will be appropriately skilled and trained. Currently there is no nurse involved in providing the FECH or any similar program to patients or carers.

**Fig. 1:**
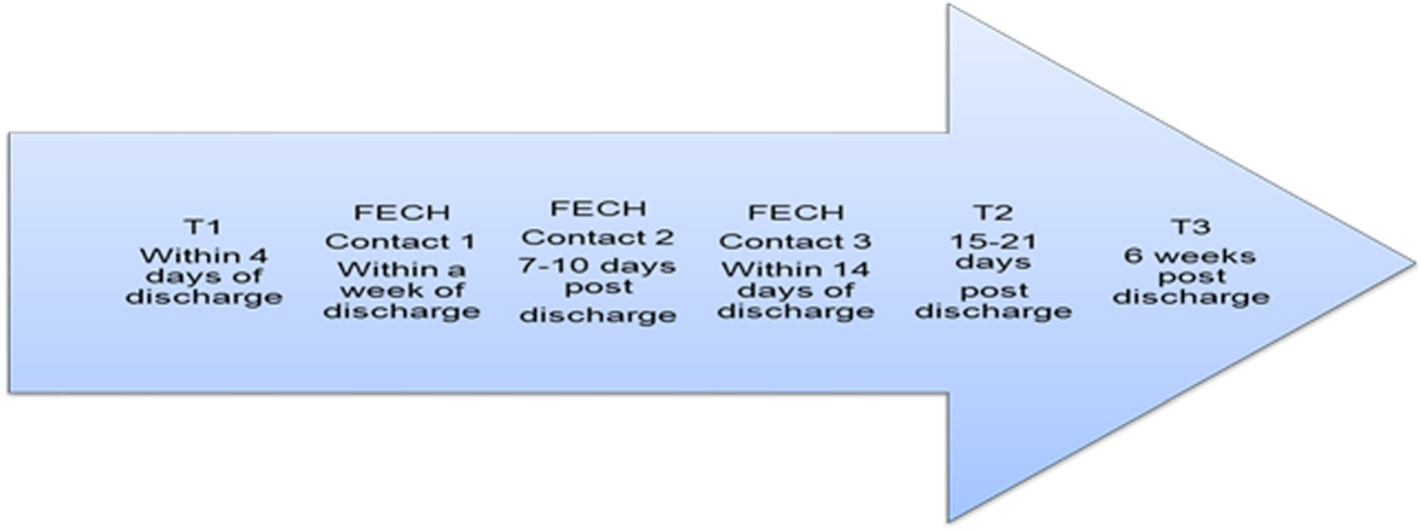
Data collection and intervention points (experimental condition, family participants)

Contacts will be made by telephone, after discharge (Fig. 1). The FECH Nurse will make initial contact with the family carer within one week of the discharge (Contact 1). This contact will include the FECH nurse introducing him/herself, explaining the intervention, and scheduling ‘Contact 2’. Contact 2 is likely to comprise a series of brief telephone interviews, if this is the arrangement that suits the family carer best, within the subsequent few days to (a) determine and respond to the extent to which the family carer understands the copy of the discharge letter to the general practitioner that has been provided to them; (b) administer the CSNAT [14] to the family carer, resulting in the carer’s self-identified and highest prioritised support needs; and (c) initiate responses to the three prioritised needs, helping ensure family carers’ linkage and engagement with appropriate existing resources. A few days after Contact 2 is finalised (within 14 days of the discharge), the FECH nurse will check to determine if access to support has been achieved as planned, advising as appropriate (Contact 3). This contact will include asking if the services have been sourced (eg, an appointment made) and/or if the service has been accessed. These three contact points will be coordinated with the three data collection time points (T1, T2, and T3) also shown in Fig. 1.

### Control condition (usual care)

Standard discharge ‘care’ includes the provision of a letter from the MAU’s physician to the patient’s general practitioner, with a copy provided to the patient. Medications are provided/organised by the MAU pharmacist. The MAU information booklet provides information about post-discharge support options and is intended to be taken home. Carers regarded as ‘at particular risk’ by the social work team receive social work assessment and links to services. Services put in place for patients may include a variety of care packages or programs. Information packs from Carers Australia are made available in the hospital rooms. Any one or more of these options considered by treating ward staff to be relevant to the patient and their carer will be provided as usual care.

### Carer outcome and data collection

#### Primary outcome variable

The primary outcome is preparedness for caregiving, to be measured with the Preparedness for Caregiving Scale (PCS) from the Family Care Inventory [19] (T1- T3). This measure is an 8-item scale, with five response options (0 = not at all prepared, 4 = very well prepared) and is designed for use with carers of older people receiving homecare or experiencing care transitions. Construct validity has been established in older people, and the measure has been shown to be reliable (Cronbach’s alpha coefficients: 0.88-0.93) [19]. Testing in patients with a life threatening illness confirmed satisfactory internal consistency reliability as well as unidimensionality [20].

#### Potentially confounding variables

Data on potentially confounding variables will be collected to help inform interpretation of results. Carer rated patient Symptom Assessment Scale scores [21] and carer rated scores evaluating dependence of the patient in ten activities (Barthel Activities of Daily Living Index) [22] will be collected (T1-T3). Robust psychometric properties have been documented for these tools. Carer resilience will be measured at T1 using the 10-item Connor-Davidson Resilience Scale [23].

#### Secondary outcome variables

Based upon recent findings using the CSNAT tool [24], caregiver strain will also be measured at each time point using the 25-item Family Appraisal of Caregiving Questionnaire [25]. Carers’ ratings of their own health will also be collected (T1-T3) using the SF12 Version 2, a tool exhibiting robust psychometric properties with older people [26]; this tool has been used in large scale surveys such as those with veterans in the United States [27].

#### Data collection

A Research Officer (RO1) will develop a schedule for collecting data from all participating family carers (both groups) by telephone, at the time of recruitment to the study. At baseline assessment (T1), RO1 will also collect demographic details of the patient and the carer from the carer, including the carer’s familial relationship with the patient, age and gender (carer and patient), highest education level, occupation, usual place of residence, country of birth, years in Australia, support provided for the patient, contact with the patient, length of time caring for the patient, education received to help in the caring role, location of care, whether currently living with patient (and if so, whether in patient’s or carer’s home), any care provided by others, and known current medical conditions (patient and carer). Brief, robust measures to minimise questionnaire burden will then be administered (each contact is estimated to last approximately 30 min). Questionnaire administration across the three assessments (T1, T2, and T3) is summarised in Table 1.

**Table 1:** Planned questionnaire administration at each time point

Time 1	Time 2	Time 3
Demographic details		
Connor-Davidson Resilience Scale [23]		
Preparedness for Caregiving Scale [19]	Preparedness for Caregiving Scale [19]	Preparedness for Caregiving Scale [19]
Symptom Assessment Scale [21] (patient)	Symptom Assessment Scale [21] (patient)	Symptom Assessment Scale [21] (patient)
Barthel Activities of Daily Living Index [22] (patient)	Barthel Activities of Daily Living Index [22] (patient)	Barthel Activities of Daily Living Index [22] (patient)
SF12 [26]	SF12 [26]	SF12 [26]
Family Appraisal of Caregiving Questionnaire [25]	Family Appraisal of Caregiving Questionnaire [25]	Family Appraisal of Caregiving Questionnaire [25]

### Patient outcomes and data collection

Quantitative patient outcome data will address Western Australian health care system utilisation and will be obtained through linked administrative health data provided via the WA Data Linkage System (WADLS) [28]. These Data Linkage Unit (DLU) data will capture reason for service and mode of transport plus: date of service, symptom/presenting problem, and triage code (ED presentation); admission and separation dates, primary diagnosis code, co-diagnosis code, and diagnosis related group (hospitalisation data). In addition, to account for time at risk, and to capture deaths resulting from acute events during the study follow-up period, date and cause of death will be collected.

The data retrieved from data linkage will cover the time period from the date of the index separation of the patient whose carer is the first to be recruited to the study until 3 months after the index separation of the patient whose carer is the last to be recruited. Since recruitment is planned to last approximately 6 months, this time period will cover approximately 9 months.

### Sample size

The primary outcome is the total score on the Preparedness for Caregiving Scale [19]. An improvement of two points in the total score is regarded as clinically relevant, given that this would mean a change such as progressing to ‘very well prepared’ from ‘well prepared’ in 25 % of items. To detect a change of this magnitude with 80 % power (assuming that the standard deviation of the change in mean score is approximately 0.5, as in previous work) [19, 29] a sample size of 63 per group will be required. Based upon previous studies by the research team, attrition of approximately 30 % is expected so a sample of 180 carers in total will be recruited.

### Recruitment and randomisation

**Fig. 2:**
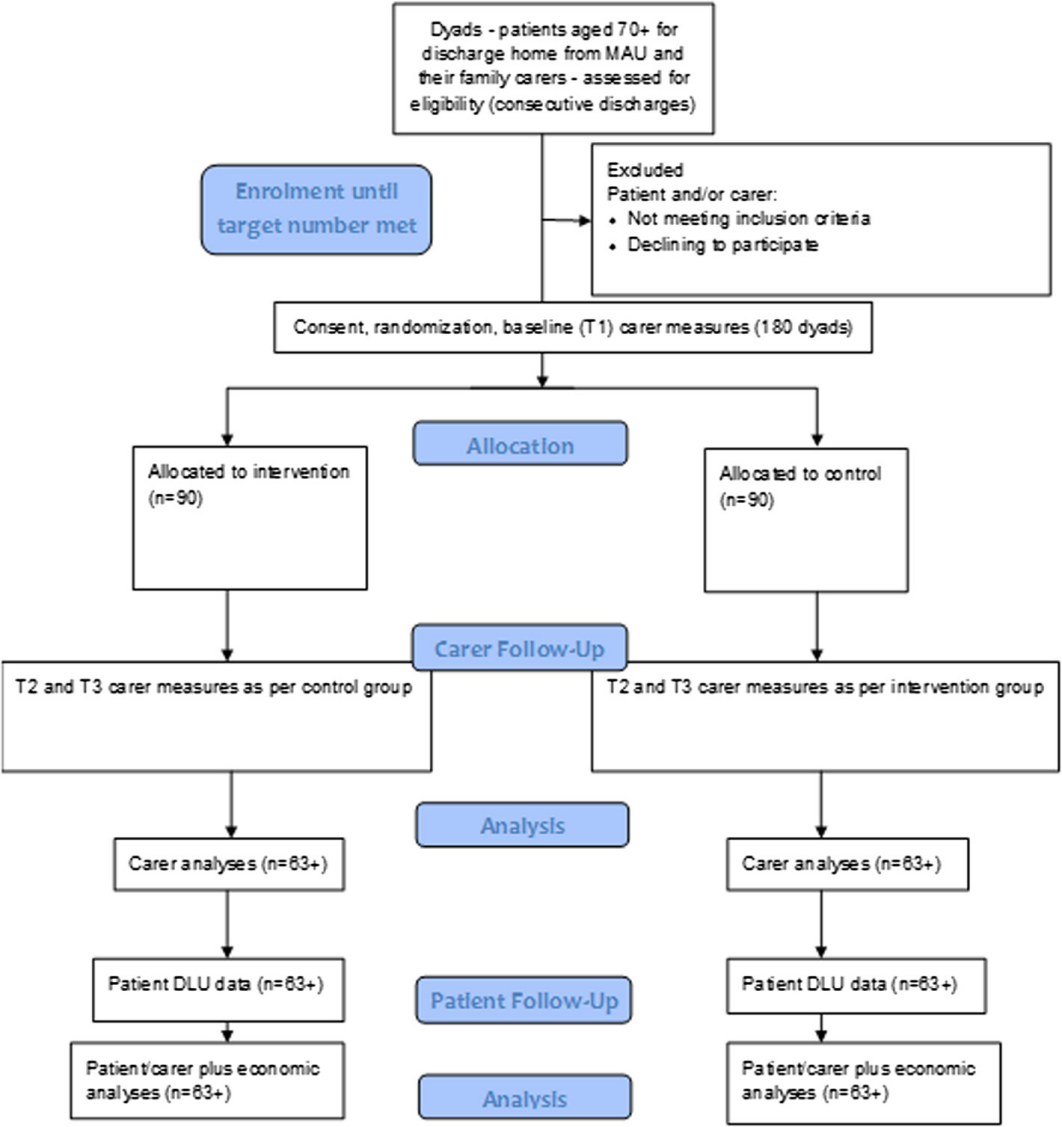
Modified consort diagram to illustrate trial study flow and participant numbers

Figure 2 shows the proposed study flow. There were more than eight MAU discharges of patients aged 70+ each day in 2013, approximately half returning home (estimated 120 per month). Therefore, recruitment of 180 participants is considered achievable within the 6 month recruitment period (30 per month).

Although the primary study outcome relates to carers of patients discharged from the MAU, other outcomes relate to the former patients themselves so carer/patient dyads will be recruited. Whenever a patient aged 70 years or older is admitted to the MAU during the recruitment period, family carers - and, when appropriate, patients - will be provided with information about the project and asked to provide written consent to participate in the study by RO1. However, many patients are likely to be too unwell to be approached with information about the study during their hospitalisation and are likely to experience ongoing poor health that may also fluctuate [9]. Others will lack (cognitive) capacity to consider consenting. In these instances, data about patients’ use of health care services will be sought via a waiver of consent. An opt out form will be provided for such patients in case there is a later opportunity – if and when the patient’s health has improved – for them to withhold consent for inclusion of their data in the study, should this be their wish.

To facilitate the assignment to group (control or intervention) of participants, a list of treatment allocations will be prepared before the study commences; it will contain a study number (whole number, starting from one), and a code to indicate the group (intervention or control). The list of codes will be obtained using a sequence of computer-generated random numbers, and organised so that recruitment to the two study arms occurs at an approximately equal rate (using a permuted random block strategy). Allocation of participants to their treatment group will occur as follows: as each new dyad is recruited to the study, the next study number will be allocated to them (next in sequence on the list), and the treatment allocation for that study number will be read from the list.

### Qualitative feedback from patients and family carers

An ‘exit’ telephone interview will be conducted with an estimated 30 carers in the intervention group and, when possible, the older people receiving care from them (estimated 20). These participants will be purposively sampled on the basis of questionnaire data to cover a wide variety of carer profiles (eg, carer sex, age [older versus younger carers], duration of caregiving [new carers versus those who are more experienced], relationship to patient [husbands, wives, sons, daughters, others], and location of care). The sample will be extended if issues need further exploration and until data saturation is reached.

Interviews will occur within two weeks following administration of the final measures, to: investigate how the FECH model affected the participants, assess the support provided, identify factors influencing feasibility/usefulness, and assess how the FECH model could be improved. Written informed consent for participation will be obtained separately for this step and will involve mailing the information sheet and consent forms (for the carer and for the patient); then following up with a phone call during which questions can be answered, before requesting the return of the completed consent form in a prepaid study envelope. It is anticipated that the interviews will take approximately 30 min. A second telephone call will be offered if the interview takes longer than 30 min and the carer wishes to provide further relevant information.

### Qualitative feedback from the hospital staff

A focus group interview will be convened with the MAU staff as soon as carer recruitment for the trial has been completed. The staff working on the unit during the trial will be invited to take part and will be asked to comment on any impact from the FECH program on the Unit and to suggest: program refinements that would minimise negative outcomes and maximise those that are positive, how the program could be integrated into practice, and how its sustainability could be addressed. The staff members will be invited to take part by mail, and will also be sent an information sheet and consent form.

### Process evaluation

Evaluation of FECH program processes will involve the FECH nurse documenting, during the trial: (a) adherence to, or deviation from, planned FECH processes; (b) information provided to carers and the resources to which they were referred; (c) the extent to which carers engaged with resources; (d) the contextual factors that were barriers to, or facilitators of, resource and service access and engagement; and (e) costs issues – primarily time taken to implement processes. This documentation will be achieved using an electronic database developed at the same time as the process manual and completed by the FECH nurse as the process is implemented.

### Blinding

Group assignment will be concealed from RO1. When RO1 conducts the T1-T3 data collection telephone questionnaire administration, he or she will commence the conversation with a request to the participant (the carer) not to mention any phone calls from other study personnel. This reminder will not always be sufficient to prevent disclosure, and instances when it occurs will be documented to inform study reports. In a previous study, quantification of any ‘unblinding’ involved asking the hospital staff to identify to which group they thought patient participants had been assigned [30]. In this study, a similar question will be asked of the research staff collecting outcome data. The person conducting the qualitative interviews will be a Research Officer employed for this purpose alone (RO2), so that blinding to group allocation will be maintained for RO1. Investigator blinding will also be maintained by allocating responsibility for this (qualitative) component of the study to one of the research team, an experienced qualitative researcher who will not be involved in quantitative data collection or analyses.

### Statistical analysis

Data analyses will be performed using the IBM SPSS statistical software package [31]. A p-value of <0.05 will be taken to indicate a statistically significant result in all tests.

#### Baseline data

Standard descriptive statistics (means, standard deviations, medians for continuous variables and frequencies and percentages for variables measured on a categorical scale) will be used to summarise the characteristics of participants in the study (primarily demographic data, but also including some data relating to the care recipient to describe the caregiving situation). Chi-square tests and t-tests will be used to compare the treatment groups (intervention versus control) on the basis of these variables. It is anticipated that there will be no significant differences between groups in terms of demographic characteristic or potentially confounding variables, which will confirm that the randomisation process has allocated participants evenly to each arm of the study.

#### Carer outcome

Outcome analysis will be conducted using an Intention to Treat approach. The change in the total Preparedness for Caregiving Scale [19] score between T1 and T3 will be calculated for each carer. An initial *t*-test will compare changes between treatment groups. If changes in scores are not normally distributed, a non-parametric method will be used instead (Wilcoxon 2-sample test). In the (unlikely) event that groups differ on the basis of baseline variables, a regression model will be used instead of the *t*-test, so that these potentially confounding variables can be taken into account. A random effects regression model will be used to examine changes in the Preparedness for Caregiving Scale score over all three data collection periods (instead of just baseline/end of study). This model will be used so that correlations between measurements made on the same participants can be taken into account. Results of the model will help show whether changes in scores happen soon after recruitment, or later. The model can be extended to adjust for other potentially confounding variables.

#### Patient outcomes

Patient outcomes will be compared between groups using t-tests or regression models. For example, the total length of hospital stay will be calculated and compared between groups using a *t*-test (after log-transformation of the data if indicated). Number of re-admissions to hospital or presentations to ED will be compared using the same method. A regression model will be used to take into account potentially confounding variables (if relevant). The association between change in preparedness and change in health care utilisation will be evaluated using carer outcomes linked to the administrative data.

### Economic costs and associated analysis

Since a randomised controlled trial is being undertaken to determine the efficacy of the new support program for older patients’ carers, the economic evaluation reflects a service substitution model without cost sharing or transfer. Costs of the program will be evaluated using prospective data collection for each patient/carer dyad (cases and controls) and will include the costs associated with the intervention and outcomes using a Western Australian health system perspective.

#### Program costs

Program costs in addition to those for usual care will include those documented during the process evaluation, in particular: (1) training of the FECH nurse to implement the program; (2) the FECH nurse salary; (3) costs associated with providing discharge information to carers (costed on a case by case basis) – FECH nurse time and web access (to identify service providers), any costs for communication that is part of the intervention, and stationery used. Costs associated with research components/activities will be excluded since they would not be incurred if the program was taken up. Outcome costs associated with utilisation of health services during follow up for patients (cases and controls) will include: (1) cost of ED visits, (2) cost of additional in-patient hospitalisation, and (3) cost of ambulance use.

#### In-patient costs

In-patient costs will be calculated using Diagnostic Related Group (DRG) based costings from the appropriate cost report available from the Australian Government Department of Health. Since Urgency Related Group (URG) is not available directly from the ED data, cost of ED attendances will be based on derivation of the closest urgency related/disposition group using data obtained from the Standard Emergency Record Information. The derivation algorithm follows as closely as possible the URG Grouper application developed by the Independent Hospital Pricing Authority (IHPA) which uses Episode End Status, Type of Visit, Triage, Sex, and Diagnosis Code. Costing will then be undertaken using the price weight of the URG and National Efficient Price provided in the closest IHPA National Efficient Price Determination report to the date of the episode [32].

#### Patient ambulance utilisation

Patient ambulance utilisation will be costed at $AUD898 per service. The use of ambulance services will be obtained via the hospital mortality data system data (source of referral transport) and ED (arrival type-transport mode) data sets.

#### Analysis

Costs and outcomes associated with delivering the intervention will be compared using cost-consequence analysis, a variant of cost-effectiveness analysis in which the components of incremental costs and outcomes are computed and listed without aggregating these results into an overall ratio. Cost-consequence analysis provides a more comprehensive presentation of information than other types of economic evaluation and is appropriate for complex interventions that generate outcomes that cannot meaningfully be expressed using a single metric such as those in this study. Consequences (outcome measures as described above under patient and carer data) and net costs (cost of intervention minus cost savings produced by the intervention) will be tabulated to allow analysis of incremental cost per net change for each outcome.

A decision tree analysis using TreeAge Pro 2015 [33] will evaluate cost-consequence separately for each outcome. Decision trees are widely used to illustrate the conceptual model of a cost effectiveness analysis. The tree begins with a decision node depicting treatment options (intervention versus usual care) for study participants. Each option becomes a main branch off the box, which further divides into smaller branches at a ‘chance node’ as certain pre-defined events (outcomes) occur. Decision trees illustrate both the probability of each outcome and the costs associated with the resultant outcome. The likelihood of each consequence is expressed as a probability of occurrence and cost calculated from trial data. Thus it will be possible to calculate expected cost and expected outcome of each option. For a given option, the expected cost is the sum of costs of each consequence weighted by the probability of that consequence.

As with all research, this study will incorporate assumptions, uncertainties, and variability in the data. To evaluate the importance of the assumptions and uncertainties, comprehensive sensitivity analyses (deterministic and probabilistic) will be performed. Sensitivity analyses strengthen economic evaluations by indicating the stability of the reported outcome and identifying which variables have the most influence on it. This step will provide an estimation of likely generalisability of the cost-consequence estimate outside the trial. Examples of uncertainties and variables are in Table 2. Where average costs are used, best and worst case scenarios will be investigated for potential impact on cost-consequence. Effectiveness variations (eg, patient risk profiles) will be considered but most model variations will be performed on cost variables.

**Table 2:** Variables to be included in the sensitivity analysis

Variable	Possible range
Rate and type of re-admission (effectiveness)	Vary according to range in the trial
Rate of ED presentation ± ambulance (effectiveness)	Vary according to range in the trial
Magnitude of change in carer preparedness (effectiveness)	Vary according to range in the trial
Features of usual care (Cost)	Vary based on type of staff included and resources provided
FECH nurse model (Cost)	Vary according to qualifications, hours required, nurse/patient load.
Number of patients requiring service (Cost)	Vary according to bed capacity.
Type of assistance required by carer (cost)	Vary phone versus face to face
Type of patient	Vary gender, age, clinical profile

### Qualitative analysis

All interviews will be audio-recorded and transcribed verbatim. Analysis will be aided by the use of the NVivo software program [34]. Responses will be coded on a question-by-question basis, codes will be collapsed into themes across questions, and quotes from participants will illustrate each theme. This qualitative analysis will be undertaken independently by the investigator responsible for overseeing qualitative evaluations and the interviewer to ensure consideration of the non-verbal context. These analysts will compare themes and sub-themes until agreement is reached.

### Analysis for process evaluation

Findings from the process evaluation will be summarised to support recommendations. In addition, a feasible ‘caseload’ for a full-time FECH nurse will be determined.

## Discussion

Increasing numbers of older people are experiencing multiple chronic health conditions, disability, and high level health care utilization [2]. Strategies are needed that minimise unnecessary hospital presentations and admissions and also focus on reducing hospital ‘length of stay’, while maintaining quality of care and outcomes for older patients and their carers. Many older people experiencing health challenges receive care from their families [4], including when discharged home from hospital. The success of these transitions home from hospital by older people is dependent on a range of factors including patient and carer factors, and health and care system factors – the carer being a critical element. Poor health is a recognised family caregiving outcome and support has been shown to help carers better manage and sustain their caregiving role [6].

This study investigates a novel approach to identifying prioritised carers’ needs in the immediate weeks post- discharge from hospital of the older person for whom they are providing informal care - evaluating a program aimed to support carers in this challenging time of re-acclimatisation to the home environment that has been shown to be associated with high risk of hospital re-admission [35]. Importantly, the model of care being investigated in this study, if shown to be effective and cost-effective, has potential to be incorporated relatively easily into existing discharge and post discharge follow-up activities. A recent systematic review found that discharge planning helped minimise use of the hospital setting but that involvement of the carer in the discharge planning process was seldom clearly articulated [36]. In contrast, this study has a central focus on systematically assessing and addressing carers’ needs.

The study also includes a comprehensive economic evaluation of the intervention, with robust service use data obtained via the comprehensive Western Australian Data Linkage System such as hospitalisation, ED use, and death and morbidity data. Results of this analysis will inform health services of the cost effectiveness associated with implementing the FECH program more widely across the health system.

In summary, this randomised controlled trial with embedded economic evaluation will determine carer, patient, and health service outcomes associated with introduction of a carer support program for carers of older patients being discharged from an acute medical unit in a tertiary hospital. The program may be appropriate for incorporation into routine post-discharge support if shown to be effective and if costs of implementation are offset by resultant cost savings.

## References

[1] Australian Bureau of Statistics. 3218.0 - Regional Population Growth, Australia, 2011–12. Canberra, ACT: Author; 2013. http://www.abs.gov.au/ausstats/abs@.nsf/Products/3218.0~2011-12~Main+Features~Western+Australia#PARALINK2. Accessed April 13 2015.

[2] Marengoni A, Angleman S, Melis R, Mangialasche F, Karp A, Garmen A, Meinow B, Fratiglioni L (2011). Aging with multimorbidity: A systematic review of the literature. Ageing Res Rev.

[3] Health Workforce Australia: Health Workforce Australia Annual Report 2012–13. Adelaide, South Australia: Author; 2013. http://www.hwa.gov.au/resources/publications. Accessed April 13 2015.

[4] Australian Government Department of Health and Ageing: Living longer living better. Canberra, ACT, Australia: Author; 2012. https://www.dss.gov.au/sites/default/files/documents/06_2014/a_-_aged_care_in_australia_is_changing.pdf. Accessed April 13 2015.

[5] Australian Government Productivity Commission: Caring for Older Australians: Productivity Commission Inquiry Report No 53. Canberra, ACT: Author; 2011. http://www.pc.gov.au/inquiries/completed/aged-care/report. Accessed April 13 2015.

[6] Australian Government Department of Health and Ageing: Guidelines for a palliative approach for aged care in the community setting: Best practice guidelines for the Australian context. Canberra, Australian Capital Territory: Author; 2011. http://www.health.gov.au/internet/main/publishing.nsf/Content/33A5C4EAB62FF446CA257BF0001C9698/$File/COMPAC%20Vol%201%20RTF%20accessible%20WEB.pdf. Accessed April 13 2015.

[7] Fox MT, Persaud M, Maimets I, Brooks D, O'Brien K, Tregunno D (2013). Effectiveness of early discharge planning in acutely ill or injured hospitalized older adults: A systematic review and meta-analysis. BMC Geriatr.

[8] Bauer M, Fitzgerald L, Haesler E, Manfrin M (2009). Hospital discharge planning for frail older people and their family. Are we delivering best practice? A review of the evidence. J Clin Nurs.

[9] Slatyer S, Toye C, Matthews A, Williamson DJ, Hill A, Popescu A (2013). Early re-presentation to hospital after discharge from an Acute Medical Unit: Perspectives of older patients, their family caregivers and health professionals. J Clin Nurs.

[10] Funk L, Stajduhar KI, Toye C, Grande G, Aoun S, Todd C (2010). Part 2: Home-based family caregiving at the end of life: A comprehensive review of published qualitative research (1998–2008). Palliat Med.

[11] Grande G, Stajduhar K, Aoun S, Toye C, Funk L, Addington-Hall J, Payne S, Todd C (2009). Supporting lay carers in end of life care: Current gaps and future priorities. Palliat Med.

[12] Stajduhar KI, Funk L, Toye C, Aoun S, Grande G, Todd C (2010). Part 1. Home-based family caregiving at the end of life: A comprehensive review of published quantitative research (1998–2008). Palliat Med.

[13] Ewing G, Grande G (2013). Development of a Carer Support Needs Assessment Tool (CSNAT) for end-of-life care practice at home: A qualitative study. Palliat Med.

[14] Ewing G, Brundle C, Payne S, Grande G (2013). The Carer Support Needs Assessment Tool (CSNAT) for use in palliative and end-of-life care at home: A validation study. J Pain Symptom Manage.

[15] Aoun S, Deas K, Toye C, Ewing G, Grande G, Stajduhar K (2015). Supporting family caregivers to identify their own needs in end-of-life care: Qualitative findings from a stepped wedge cluster trial. Palliat Med.

[16] Moher D, Hopewell S, Schulz KF, Montori V, Gøtzsche PC, Devereaux PJ, Elbourne D, Egger M, Altman DG (2010). CONSORT 2010 Explanation and elaboration: Updated guidelines for reporting parallel group randomised trials. J Clin Epidemiol.

[17] Scott I, Vaughan L, Bell D (2009). Effectiveness of acute medical units in hospitals: A systematic review. Int J Qual Health Care.

[18] Aggar C, Ronaldson S, Cameron ID (2011). Self-esteem in carers of frail older people: Resentment predicts anxiety and depression. Aging Ment Health.

[19] Archbold PG, Stewart BJ, Greenlick MR, Harvath T (1990). Mutuality and preparedness as predictors of caregiver role strain. Res Nurs Health.

[20] Hudson PL, Hayman-White K, Measuring the psychosocial characteristics of family caregivers of palliative care patients: Psychometric properties of nine self-report instruments. J Pain Symptom Manage 2006. doi: 10.1016/jpainsymman.2005.07.010. 10.1016/j.jpainsymman.2005.07.010 16563316

[21] Aoun S, Monterosso L, Kristjanson K, McConigley R (2011). Measuring symptom distress in palliative care: Psychometric properties of the Symptom Assessment Scale (SAS). Palliat Med.

[22] Collin C, Wade D, Davies S, Horne VT (1988). The Barthel ADL Index: A reliability study. Int Disabil Stud.

[23] Connor KM, Davidson JRT (2003). Development of a new resilience scale: The Connor-Davidson Resilience Scale (CD-RISC). Depress Anxiety.

[24] Aoun SM, Grande G, Howting D, Deas K, Toye C, Troeung L, Stajduhar K, Ewing G. The impact of the Carer Support Needs Assessment Tool (CSNAT) in community palliative care using a stepped wedge cluster trial. PLoS One. 2015; e0123012. doi:10.1371/journal.pone.0123012. 10.1371/journal.pone.0123012 PMC4388632 25849348

[25] Cooper B, Kinsella GJ, Picton C (2006). Development and initial validation of a family appraisal of caregiving questionnaire for palliative care. Psychooncology.

[26] Ware JE, Kosinski M, Keller SD (1996). A 12-item Short Form Health Survey:Construction of scales and preliminary tests of reliability and validity. Med Care.

[27] Der-Martirosian C, Cordasco K, Washington D (2013). Health-related quality of life and comorbidity among older women veterans in the United States. Qual Life Res.

[28] Holman CD, Bass AJ, Rouse LL, Hobbs MST (1999). Population-based linkage of health records in Western Australia: Development of a health services research linked database. Aust N Z J Public Health.

[29] Schumacher KL, Stewart BJ, Archbold PG, Caparro M, Mutale F, Agrawal S (2008). Effects of caregiving demand, mutuality, and preparedness on family caregiver outcomes during cancer treatment. Oncol Nurs Forum.

[30] Haines T, Bennell K, Osborne R, Hill K (2004). Effectiveness of a targeted falls prevention program in a sub-acute hospital setting: A randomised controlled trial. Br Med J.

[31] IBM Corp. Released 2013. IBM SPSS Statistics for Windows, Version 22.0. Armonk, NY: IBM Corp.

[32] Independent Hospital Pricing Authority: National pricing model technical specifications 2015–16: National Pricing Model Version 1.0. Commonwealth of Australia; 2015. http://www.ihpa.gov.au/internet/ihpa/publishing.nsf/content/3414216C0D3EDFDACA257DF60007055A/$File/National%20Pricing%20Model%20Technical%20Specifications%202015-16.pdf. Accessed April 13 2015.

[33] TreeAge Software Inc. MA, USA; 2015.

[34] NVivo qualitative data analysis software; QSR International Pty Ltd. Version 10, 2012.

[35] Iloabuchi TC, Mi D, Tu W, Counsell SR (2014). Risk factors for early hospital readmission in low-income elderly adults. J Am Geriatr Soc.

[36] Shepperd S, Lannin NA, Clemson LM, McCluskey A, Cameron ID, Barras SL: Discharge planning from hospital to home. Cochrane Database Syst Rev. 101002/14651858CD000313pub4 2013, 1(CD000313). 10.1002/14651858.CD000313.pub4 23440778

